# Supplementary figures and images for: Ultrasonographic modeling of lung and diaphragm mechanics: clinical trial of a novel non-invasive method to evaluate pre-operative pulmonary function
Source: PeerJ. 2024 Dec 19;12:e18677. doi: 10.7717/peerj.18677 (PMC11663399; doi:10.7717/peerj.18677)

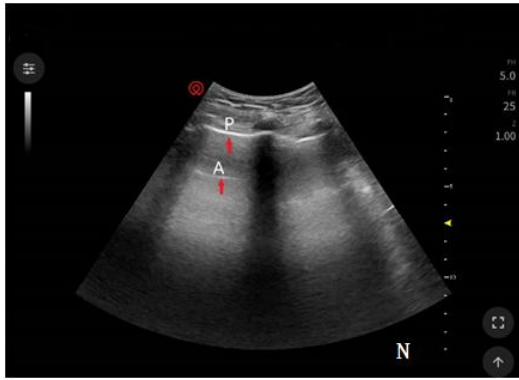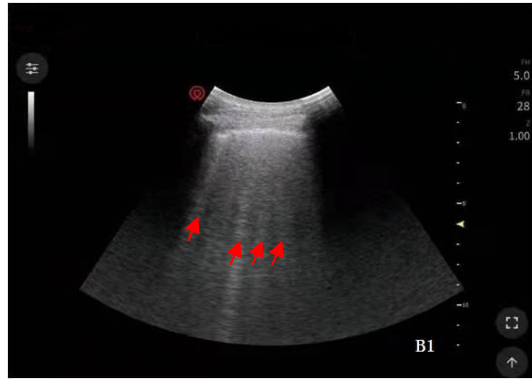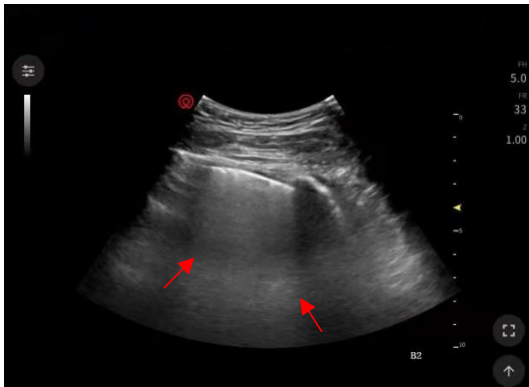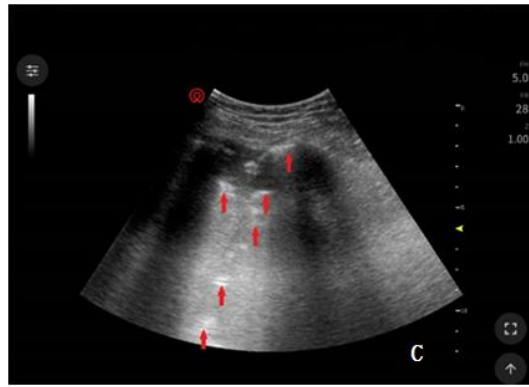

Supplement: Supplemental Information 1 — Normal aeration (N): lung sliding sign and A lines or less than 3 isolated B line(s); Moderate lung aeration loss (B1): multiple, well-defined B-lines or one or more small subpleural consolidations; Severe lung aeration loss (B2): multiple coalescent B-lines that occupy the whole lung image or multiple small subpleural consolidations; Complete aeration loss (C): localized sonographic consolidation (subpleural tissue-like pattern). [file peerj-12-18677-s001.pdf]

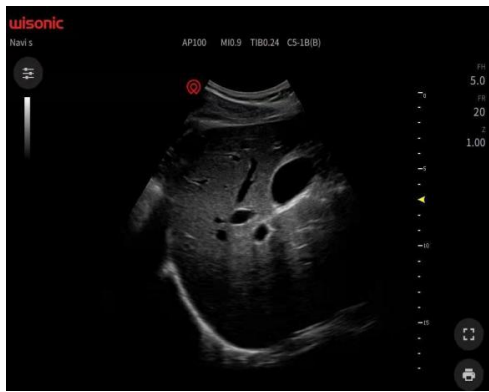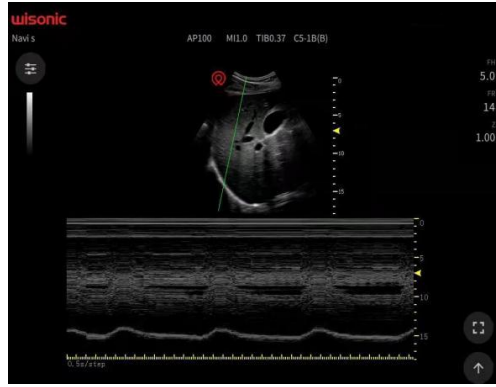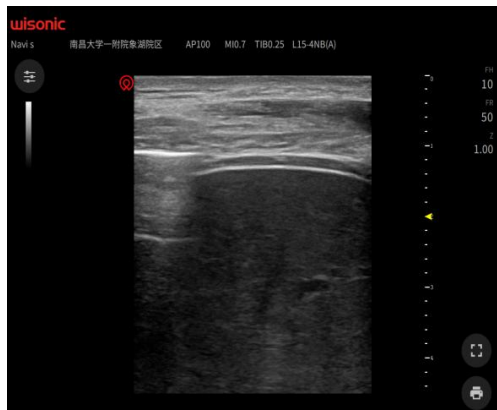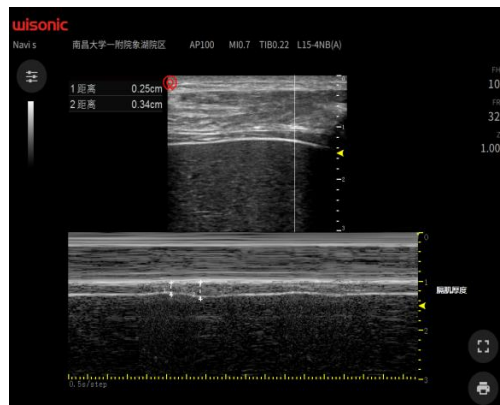

Supplement: Supplemental Information 2 [file peerj-12-18677-s002.pdf]
