# Supplementary material for: Ultrasonographic modeling of lung and diaphragm mechanics: clinical trial of a novel non-invasive method to evaluate pre-operative pulmonary function
Source: PeerJ. 2024 Dec 19;12:e18677. doi: 10.7717/peerj.18677 (PMC11663399; doi:10.7717/peerj.18677)
Supplement: Supplemental Information 5 [file peerj-12-18677-s005.docx]

**Trial Protocol**

### **Project summary**

**Rationale** Pulmonary function testing (PFT) plays a key role in assessing functional disabilities during the perioperative period. However, PFT requires the subject and examiner to cooperate and the results are influenced by both technical and personal factors. The use of ultrasound (US) for structural and functional assessments of the lungs and diaphragm, which only requires a minimum cooperation of patient, is increasing. Patients' dyspnea is mainly caused by functional lung disability but may also be caused by weak respiratory muscles. The diaphragm is a primary respiratory muscle. At present, the role of lung ultrasound (LUS) combined with diaphragmatic ultrasound (DUS) remains to be explored.

**Objectives** This study aimed to introduce a novel approach for assessing lung function using a mathematical model based on LUS and DUS.

**Methods and populations** This prospective study was performed at the First Affiliated Hospital of Nanchang University. between June 2021 and December 2021, 208 patients were recruited and underwent PFT, LUS, and DUS examinations. An experienced physician, blinded to the clinical history and PFT results, performed LUS and DUS and explored the correlations between a mathematical model (ultrasonographic modeling score [U-score]) using LUS combined with DUS and pulmonary function parameters. Univariate, multivariate, and logistic regression analyses were also performed.

**Time frame**  The trial was applied to the ethics committee of first affiliated hospital of Nanchang university and registered at chictr.org.cn from June 2021 to December 2021.

**Expected outcomes** The U-score using both lung and diaphragm ultrasound measurements may help estimate pulmonary function, thereby allowing the assessment of lung function in patients who may not be able to reliably participate in PFT.

### **General information**

- **Protocol title, protocol identifying number (if any), and date.**

Ultrasonographic modeling of the lungs and diaphragm: A novel approach to assess pulmonary function in preoperative patients from June 2021 to December 2021.

- **Name and address of the sponsor/funder.**

Name: Health Commission of Jiangxi Province (202210389).

Address:No. 72, Yuzhang Road, Donghu District, Nanchang, Jiangxi Province, China

- **Name and title of the investigator(s) who is (are) responsible for conducting the research, and the address and telephone number(s) of the research site(s), including responsibilities of each.**

Name: Tian-yuan Li^1^, Xiong-zhi Wu^2^, Ding-de Long^1^, Huan Fu^1^, Su-ping Guo^3^, Fen Liu^4#^

^1^Department of Anesthesiology, The First Affiliated Hospital ,Jiangxi Medical College, Nanchang University, NO. 1519, Dongyue Avenue, Nanchang, Jiangxi, China, 330006;

^2^Department of Anesthesiology, Shaoxing People's Hospital, NO.568, North Zhongxing Road, Shaoxing, Zhejiang, China;

^3^Department of Ultrasound Medicine, The First Affiliated Hospital ,Jiangxi Medical College, Nanchang University, NO. 1519, Dongyue Avenue, Nanchang, Jiangxi, China, 330006;

^4^Department of Intensive Care Unit, The First Affiliated Hospital ,Jiangxi Medical College, Nanchang University, NO. 1519, Dongyue Avenue, Nanchang, Jiangxi, China, 330006;

^#^ Corresponding author:

Fen Liu^4^, NO. 1519, Dongyue Avenue, Nanchang, Jiangxi, China, 330006;

Email address: liufenzzyx@163.com.

**Responsibilities:**

Tian-yuan Li: Study design, Methodology, Writing first draft, Patient recruitment, Writing-Reviewing and Editing; Xiong-zhi Wu: Data collection, Data analysis, Writing first draft; Ding-de Long: Software , Writing- Original draft preparation. Su-ping Guo: Visualization, Data collection; Huan Fu:Data collection; Fen Liu: Study design, Methodology.

- **Name(s) and address(es) of the clinical laboratory(ies) and other medical and/or technical department(s) and/or institutions involved in the research**

### Department of Anesthesiology, The First Affiliated Hospital ,Jiangxi Medical College, Nanchang University, NO. 1519, Dongyue Avenue, Nanchang, Jiangxi, China, 330006;

### **Rationale & background information**

Pulmonary function testing (PFT) is an important part of perioperative patient assessment and clinically assesses the functional impairment [1]. [preoperative PFT could be a useful marker for predicting](https://pubmed.ncbi.nlm.nih.gov/28879570/) postoperative complications [2]. In particular, forced expiratory volume in the first second/forced vital capacity (FEV1/FVC), one of the important indicators of PFT, is <60% [3]. However, PFT is limited by the patient’s ability to fully understand the basics of the test and cooperate with the examiner [4]. Thus, the challenge is to find an alternative method to assess lung function and overcome these limitations. The aim of this study was to develop objective criteria to identify patients at high risk of pulmonary dysfunction.

Ultrasound (US) is a viable and non-invasive assessment instrument that only requires a minimum cooperation of patient, making it a promising tool for clinical applications.

Lung ultrasonography (LUS) is a valuable diagnostic tool that can complement physical examinations in various respiratory diseases and settings [5]. Its ability to detect changes in both the lung parenchyma and pleural cavities renders it widely accessible. Numerous studies have indicated that LUS has significant benefits in the diagnosis of various respiratory diseases, including acute respiratory distress syndrome [6], pulmonary edema [7], interstitial lung disease [8], pneumothorax [9], atelectasis [10], pneumonia [11], and others. In addition, LUS is valuable for diagnosing and assessing lung diseases in specific populations, including newborns [11], children [12], and pregnant females [13]. LUS can serve as a key diagnostic and evaluation tool for various respiratory conditions.

# Diaphragm ultrasonography (DUS) is widely used for assessing diaphragmatic activity. Some studies have suggested an association between the ultrasound indices of the diaphragm and lung function parameters in various diseases, including osteoporosis, vertebral fractures, kyphosis, neuromuscular disease[14], patients with stroke [15],COPD [16]and healthy subjects [17].

US measurements of the thickness and thickening fraction of the diaphragm have been validated to assess diaphragmatic function [18-19].

### **References (of literature cited in preceding sections)**

1. Ruppel GL, Enright PL. Pulmonary function testing. Respir Care. 2012 Jan;57(1):165-75. doi: 10.4187/respcare.01640. PMID: 22222135.
2. Matsumi A, Takenaka R, Ando C, Sato Y, Takei K, Yasutomi E, Okanoue S, Oka S, Kawai D, Kataoka J, Takemoto K, Tsugeno H, Fujiki S, Kawahara Y. Preoperative Pulmonary Function Tests Predict Aspiration Pneumonia After Gastric Endoscopic Submucosal Dissection. Dig Dis Sci. 2017 Nov;62(11):3084-3090. doi: 10.1007/s10620-017-4750-4. Epub 2017 Sep 6. PMID: 28879570.
3. Zhang WH, Chen XZ, Yang K, Liu K, Guo DJ, Wang W, Zhang B, Chen ZX, Chen JP, Zhou ZG, Hu JK. Risk Factors and Survival Outcomes for Postoperative Pulmonary Complications in Gastric Cancer Patients. Hepatogastroenterology. 2015 May;62(139):766-72. PMID: 26897970.
4. Tojo N. [Problems of lung function testing in the laboratory]. Rinsho Byori. 2006 Aug;54(8):834-7. Japanese. PMID: 16989403.
5. Volpicelli G, Elbarbary M, Blaivas M, Lichtenstein DA, Mathis G, Kirkpatrick AW, Melniker L, Gargani L, Noble VE, Via G, Dean A, Tsung JW, Soldati G, Copetti R, Bouhemad B, Reissig A, Agricola E, Rouby JJ, Arbelot C, Liteplo A, Sargsyan A, Silva F, Hoppmann R, Breitkreutz R, Seibel A, Neri L, Storti E, Petrovic T; International Liaison Committee on Lung Ultrasound (ILC-LUS) for International Consensus Conference on Lung Ultrasound (ICC-LUS). International evidence-based recommendations for point-of-care lung ultrasound. Intensive Care Med. 2012 Apr;38(4):577-91. doi: 10.1007/s00134-012-2513-4. Epub 2012 Mar 6. PMID: 22392031.
6. Stefanidis K, Dimopoulos S, Tripodaki ES, Vitzilaios K, Politis P, Piperopoulos P, Nanas S. Lung sonography and recruitment in patients with early acute respiratory distress syndrome: a pilot study. Crit Care. 2011 Aug 4;15(4):R185. doi: 10.1186/cc10338. PMID: 21816054; PMCID: PMC3387628.
7. Gargani L, Frassi F, Soldati G, Tesorio P, Gheorghiade M, Picano E. Ultrasound lung comets for the differential diagnosis of acute cardiogenic dyspnoea: a comparison with natriuretic peptides. Eur J Heart Fail. 2008 Jan;10(1):70-7. doi: 10.1016/j.ejheart.2007.10.009. PMID: 18077210.
8. Pitsidianakis G, Vassalou EE, Vasarmidi E, Bolaki M, Klontzas ME, Xirouchaki N, Georgopoulos D, Karantanas AH, Tzanakis N, Antoniou KM. Performance of Lung Ultrasound for Monitoring Interstitial Lung Disease. J Ultrasound Med. 2022 May;41(5):1077-1084. doi: 10.1002/jum.15790. Epub 2021 Jul 22. PMID: 34291845.
9. Laursen CB, Pietersen PI, Jacobsen N, Falster C, Juul AD, Davidsen JR. Lung ultrasound assessment for pneumothorax following transbronchial lung cryobiopsy. ERJ Open Res. 2021 Jul 26;7(3):00045-2021. doi: 10.1183/23120541.00045-2021. PMID: 34322546; PMCID: PMC8311132.
10. Monastesse A, Girard F, Massicotte N, Chartrand-Lefebvre C, Girard M. Lung Ultrasonography for the Assessment of Perioperative Atelectasis: A Pilot Feasibility Study. Anesth Analg. 2017 Feb;124(2):494-504. doi: 10.1213/ANE.0000000000001603. PMID: 27669555.
11. Ma HR, Deng BY, Liu J, Jiang P, Xu YL, Song XY, Li J, Huang LH, Bao LY, Shan RY, Fu W. Lung ultrasound to diagnose infectious pneumonia of newborns: A prospective multicenter study. Pediatr Pulmonol. 2023 Jan;58(1):122-129. doi: 10.1002/ppul.26168. Epub 2022 Oct 6. PMID: 36169007
12. Amatya Y, Russell FM, Rijal S, Adhikari S, Nti B, House DR. Bedside lung ultrasound for the diagnosis of pneumonia in children presenting to an emergency department in a resource-limited setting. Int J Emerg Med. 2023 Jan 9;16(1):2. doi: 10.1186/s12245-022-00474-w. PMID: 36624366; PMCID: PMC9828356.
13. Piccolo CL, Liuzzi G, Petrone A, Fusco N, Blandino A, Monopoli F, Antinori A, Girardi E, Vallone G, Brunese L, Ianniello S. The role of Lung Ultrasound in the diagnosis of SARS-COV-2 disease in pregnant women. J Ultrasound. 2023 Jun;26(2):497-503. doi: 10.1007/s40477-022-00745-5. Epub 2022 Dec 27. PMID: 36574192; PMCID: PMC9793376.
14. DePalo VA, McCool FD. Respiratory muscle evaluation of the patient with neuromuscular disease. Semin Respir Crit Care Med. 2002 Jun;23(3):201-9. doi: 10.1055/s-2002-33028. PMID: 16088612.
15. Chen Y, Zhou S, Liao L, He J, Tang D, Wu W, Wang K. Diaphragmatic ultrasound can help evaluate pulmonary dysfunction in patients with stroke. Front Neurol. 2023 Apr 18;14:1061003. doi: 10.3389/fneur.2023.1061003. PMID: 37144002; PMCID: PMC10151578.
16. Topcuoğlu C, Yümin ET, Hizal M, Konuk S. Examination of diaphragm thickness, mobility and thickening fraction in individuals with COPD of different severity. Turk J Med Sci. 2022 Aug;52(4):1288-1298. doi: 10.55730/1300-0144.5435. Epub 2022 Aug 10. PMID: 36326387; PMCID: PMC10388057.
17. Cardenas LZ, Santana PV, Caruso P, Ribeiro de Carvalho CR, Pereira de Albuquerque AL. Diaphragmatic Ultrasound Correlates with Inspiratory Muscle Strength and Pulmonary Function in Healthy Subjects. Ultrasound Med Biol. 2018 Apr;44(4):786-793. doi: 10.1016/j.ultrasmedbio.2017.11.020. Epub 2018 Jan 17. PMID: 29373153.
18. Tralhão A, Cavaleiro P, Arrigo M, Lopes JP, Lebrun M, Rivas-Lasarte M, Le Pimpec-Barthes F, Latrémouille C, Achouh P, Pirracchio R, Cholley B. Early changes in diaphragmatic function evaluated using ultrasound in cardiac surgery patients: a cohort study. J Clin Monit Comput. 2020 Jun;34(3):559-566. doi: 10.1007/s10877-019-00350-8. Epub 2019 Jul 5. PMID: 31278543; PMCID: PMC7223646.
19. Unal O, Arslan H, Uzun K, Ozbay B, Sakarya ME. Evaluation of diaphragmatic movement with MR fluoroscopy in chronic obstructive pulmonary disease. Clin Imaging. 2000 Nov-Dec;24(6):347-50. doi: 10.1016/s0899-7071(00)00245-x. PMID: 11368935.

### **Study goals and objectives**

The evaluation of pulmonary function included both lung and respiratory muscle evaluations. Neither LUS nor DUS can fully assess pulmonary function. To date, no correlation has been demonstrated between pulmonary function indices and the combination of LUS and DUS in patients. The primary objective of our observational study was to describe LUS combined with DUS patterns in a cohort of perioperative patients and explore the correlations between these discoveries and PTF.

### **Study design**

In the prospective cross-sectional study, the enrollment and allocation of participants are compiled in Figure 1.


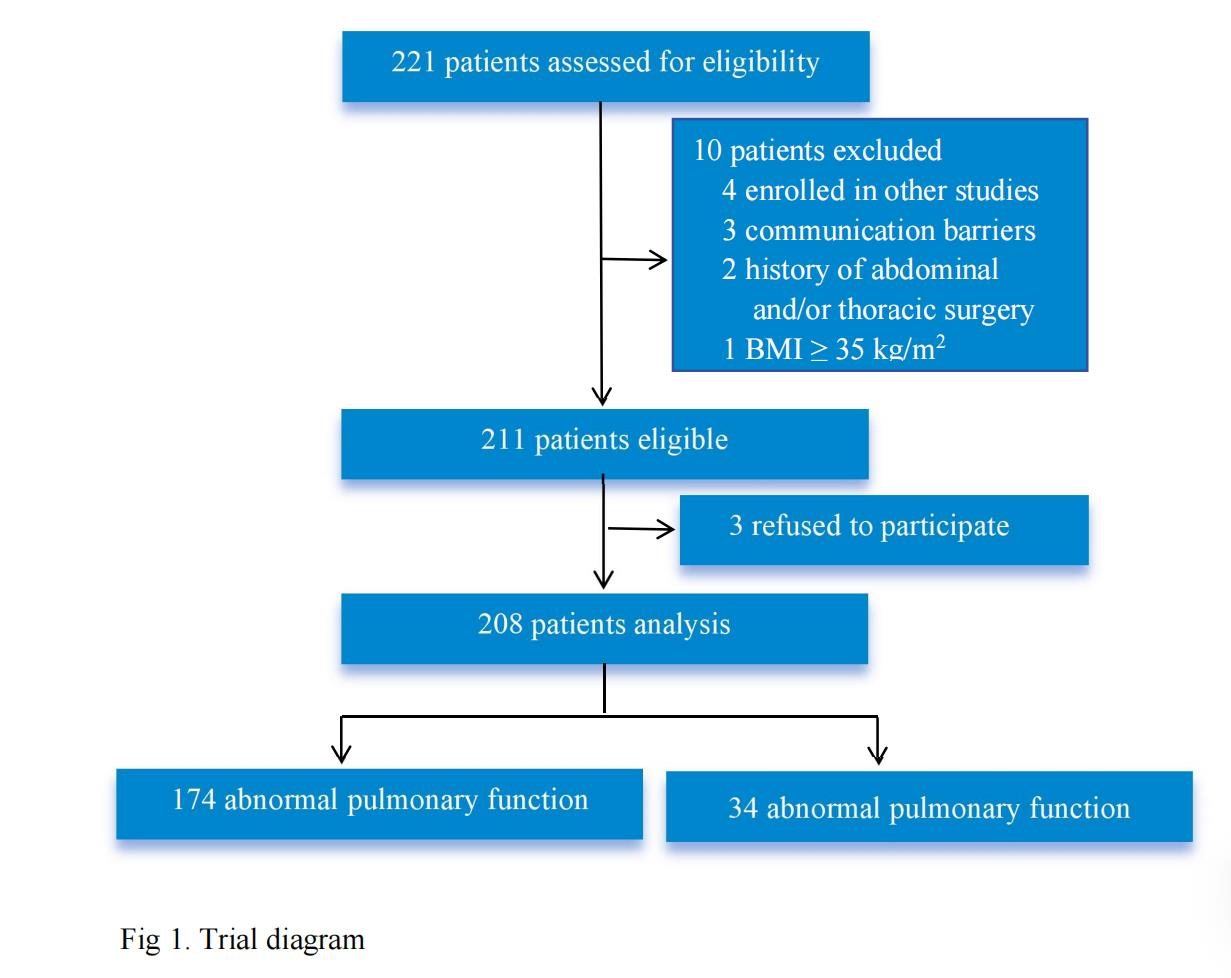


**Inclusion and exclusion criteria**

Patients aged >18 years who underwent elective surgery and consented to undergo PFT, LUS, and DUS were included. The exclusion criteria included patient decline, emergency surgery, body mass index exceeding 35 kg/m^2^, history of abdominal and/or thoracic surgery, and thoracic deformities or scoliosis.

### **Methodology**

**Pulmonary function test**

All patients underwent standard pulmonary function testing using a MasterScreen diagnostic spirometer. FEV1, forced vital capacity (FVC), and FEV1/FVC ratios were measured. According to the statement of the European Respiratory Society/American Thoracic Society (ERS/ATS) [20], FEV1/FVC<70% is the criterion for irreversible lung function impairment, whereas >70% is considered normal pulmonary function.

**Lung ultrasonography**

Patients underwent an LUS examination in accordance with a 12-zone protocol [21]， which was performed using a wisonic ultrasound machine (Wisonic Medical Co., Ltd., Shenzhen, China) by trained sonographers (T.Y.L., X.Z.W, and D.D.L.); intercostal space scanning was performed in each quadrant, and the corresponding ultrasonographic images were stored for further analysis. Lung aeration was assessed based on ultrasound images, according to previously described criteria [22]. A score of 0–3 was assigned to each quadrant by LUS, and the total LUS score was calculated by summing all 12 zones.

**Diaphragmatic ultrasonography**

A semi-recumbent position of 30° was used for all the participants. The diaphragm was scanned using a wisonic ultrasound machine (Wisonic Medical Co., Ltd., Shenzhen, China) between the anterior axillary line and midclavicular line by placing a 2–5 MHz curvilinear probe in the right subcostal region (in relation to the left side, where splenic density and gastric or intestinal gas interfere with imaging [23-25]. Diaphragm thickness (DT) was measured using B-mode US over the opposition zone of the diaphragm from the pleural line to the peritoneal line during deep breathing (D-DT) and quiet breathing (Q-DT). To determine diaphragm function, the diaphragm thickness fraction (DTF) was calculated by measuring the ability of the diaphragm to contract according to the following formula: DTF = (DT at the end of inspiration–DT at the end of expiration)/DT at the end of expiration × 100%. Diaphragm excursion (DE) was measured during both deep breathing (D-BE) and quiet breathing (Q-BE) with the sampling line and diaphragm as vertically as possible (at least 70° [26]) in M-mode. Subsequently, position markers were placed on the skin surface, three measurements were obtained, and the average was recorded.

### **Statistical analysis**

Statistical analyses were performed using SPSS version 23 software (SPSS Inc.). Continuous data were presented as the mean ± SD. Continuous data were analyzed using ANOVA or Kruskal-Wallis test. Categorical data are presented as frequencies or percentages and compared using the chi-square or Fisher’s exact test, as appropriate. Statistical analyses were performed using SPSS version 23 software (SPSS Inc.). Continuous data were expressed as the mean ± SD or median [IQR]. Continuous variables were analyzed using ANOVA or the Kruskal-Wallis test. Categorical data were expressed as frequencies or percentages. Chi-square or Fisher's exact test was used for comparison, as appropriate. Univariate logistic regression analysis was performed to identify the risk factors for abnormal pulmonary function. All variables associated with abnormal lung function, with P-value < 0.1 were candidates for backward stepwise multivariate analysis to identify independent risk factors. To predict abnormal lung function, a mathematical model was constructed using logistic regression algorithms to screen risk factors related to abnormal pulmonary function. the Hosmer-Lemeshow goodness of fit test, P < 0.05 was considered statistically significant and the degree of fit was considered good. The performance of the prediction model was evaluated in terms of sensitivity, specificity, accuracy, and area under the curve (AUC), receiver operating characteristic (ROC) analyses were performed to determine the best cut-off values to detect the outcome. P < 0.05 was considered statistically significant.

### **Expected outcomes of the study**

With validation, the U-score using both lung and diaphragm ultrasound measurements may help estimate pulmonary function, thereby allowing the assessment of lung function in patients who may not be able to reliably participate in PFT.

### **Duration of the project**

### The trial was applied to the ethics committee of first affiliated hospital of Nanchang university and registered at chictr.org.cn from June 2021 to December 2021.This prospective observational study was performed at the First Affiliated Hospital of Nanchang University, China, from 6/2021, 12/2021

### **Project management**

Tian-yuan Li: Study design, Methodology, Writing first draft, Patient recruitment, Writing-Reviewing and Editing; Xiong-zhi Wu: Data collection, Data analysis, Writing first draft; Ding-de Long: Software , Writing- Original draft preparation. Su-ping Guo: Visualization, Data collection; Huan Fu:Data collection; Fen Liu: Study design, Methodology.

### **Ethics**

### This study was approved by the Ethics Committee of the First Affiliated Hospital of Nanchang University (No. 2021-8-001).

### **Informed consent forms**

Written informed consent was obtained from all subjects participating in the trial.
